# Infant Feeding Practices of HIV Positive Mothers and Its Association with Counseling and HIV Disclosure Status in Ethiopia: A Systematic Review and Meta-Analysis

**DOI:** 10.1155/2019/3862098

**Published:** 2019-08-01

**Authors:** Getaneh Mulualem Belay, Chalachew Adugna Wubneh

**Affiliations:** Department of Pediatrics and Child Health Nursing, College of Medicine and Health Sciences, University of Gondar, Ethiopia

## Abstract

**Introduction:**

Breastfeeding is the ideal food source for all newborns globally. However, in the era of Human Immune Deficiency Virus (HIV) infection, feeding practice is a challenge due to mother-to-child HIV transmission. Therefore, this systematic review and meta-analysis aimed to estimate the national prevalence of exclusive breastfeeding and mixed feeding practices among HIV positive mothers and its association with counseling and HIV disclosure status to the spouse in Ethiopia.

**Methods:**

We searched all available articles from the electronic databases including PubMed, EMBASE, Google Scholar, and the Web of Science. Moreover, reference lists of the included studies and the Ethiopian institutional research repositories were used. Searching of articles was limited to the studies conducted in Ethiopia and published in English language. We have included observational studies including cohort, cross-sectional, and case-control studies. The weighted inverse variance random effects model was used. The overall variations between studies were checked through heterogeneity test (I^2^). Subgroup analysis by region was conducted. To assess the quality of the study, the Joanna Briggs Institute (JBI) quality appraisal criteria were employed. Publication bias was checked with the funnel plot and Egger's regression test.

**Result:**

A total of 18 studies with 4,844 participants were included in this study. The national pooled prevalence of exclusive breastfeeding and mixed feeding practices among HIV positive mothers were 63.43% (95% CI: 48.19, 78.68) and 23.11% (95% CI: 10.10, 36.13), respectively. In the subgroup analysis, the highest prevalence of exclusive breastfeeding practice was observed in Tigray (90.12%) and the lowest in Addis Ababa (41.92%). Counseling on feeding option with an odds ratio of 4.32 (95% CI: 2.75, 6.77) and HIV disclosure status to the spouse with an odds ratio of 6.05 (95% CI: 3.03, 12.06) were significantly associated with exclusive breast feedings practices.

**Conclusion:**

Most mothers report exclusive breastfeeding, but there are still almost a quarter of mothers who mix feed. Counseling on feeding options and HIV disclosure status to the spouse should be improved.

## 1. Introduction

About 90% of pediatrics HIV infection is Mother-To-Child Transmission (MTCT), which may occur during pregnancy, delivery, and breastfeeding [1]. The estimated risk of HIV transmission for nonusers of Antiretroviral Therapy (ART) was during breastfeeding (5-20%), overall without breastfeeding (15-25%), overall with breastfeeding to six months (20-35%), and overall with breastfeeding to 18-24 months ranging from 30 to 45% [2, 3]. In the recent studies related to the use of antiretroviral (ARV) therapy, reducing the risk of HIV transmission through breastfeeding showed a promising effect [4].

Breastfeeding especially in the first 12 months of life can significantly prevent malnutrition, infectious diseases, and mortality compared to nonbreastfed infants [5, 6]. In low- and middle-income countries, exclusive breastfeeding practice was considered as a major prevention factor for malnutrition [7]. Avoiding breastfeeding can eliminate the risk of mother-to-child HIV transmission in the postnatal period, but mixed and replacement feeding practices were associated with increased infant mortality and morbidity in sub-Saharan Africa countries [8].

According to the World Health Organization (WHO) guideline, breastfeeding, especially early initiation, and exclusive breastfeeding were the most critical factors in improving child survival. Moreover, the WHO's global public health direction for all infants is to breastfeed exclusively (EBF) for the first six months and then introduce nutritionally adequate and safe complementary foods while breastfeeding continues for up to 2 years of age or beyond [9, 10]. However, the WHO criteria are rarely met in developing countries and mixed feeding (MF) is common [11, 12]. This may be extremely difficult in case of HIV infected mother [13].

Therefore, the decision on infant feeding practice in the era of HIV is a big challenge for caretakers and health care providers [11, 14]. Even though exclusive breastfeeding is the best choice of feeding option in the first 6 months of the postnatal period, mother-to-child HIV transmission through breastfeeding is a major concern [14, 15]. Such a dilemma can be addressed through effective counseling and by disclosing their HIV status to their families [16, 17]. Counseling on the use of ART, adherence to ART, mother-to-child HIV transmission, feeding option, and importance of disclosing HIV status could enhance the decision on infant feeding options [18–20].

In Ethiopia, studies on infant feeding practice and its predictors among HIV positive mothers have been conducted in different parts of the country with a time variation. But, the results of these studies were inconsistent. Therefore, the aim of this systemic review and meta-analysis is to estimate the national pooled prevalence of exclusive breastfeeding and mixed feeding practices among HIV positive mothers and of its association with counseling on feeding option and HIV status disclosure to the spouse in Ethiopia.

## 2. Methods

### 2.1. Protocol Registration

The protocol of this systematic review and meta-analysis has been registered in the International prospective register of systematic review and meta-analysis (Prospero) with a registration number of CRD42018110574.

### 2.2. Reporting

For reporting of the findings, the Preferred Reporting Items for Systematic Review and Meta-Analysis (PRISMA) guideline was employed (Additional file 1).

### 2.3. Databases and Searching Strategies

All available articles were searched in PubMed, Google Scholar, EMBASE, and the Web of Science. Moreover, studies were searched from the reference lists of included studies and the Ethiopian institutional research repositories. Searching was employed using the following searching terms including “infant”, “neonate”, “child”, “infant feedings”, “Infant feeding practices”, “child feeding practices”, “exclusive breastfeeding practices”, “mixed feeding practices”, “HIV”, “Human Immune Deficiency Virus”, “Mother”, “HIV positive mothers”, “Human Immune Deficiency Virus Positive mothers”, “HIV infected mothers”, “HIV exposed infants”, “HIV infected infants”, “predictors”, “factors”, “ barriers”, “risk factors”, “prevalence”, and “Ethiopia”. The search string was developed using “AND” and “OR” Boolean operators. The searching was done from September 10 to October 28/2018. As for the search of articles in PubMed, we used this searching string (Additional file 2).

### 2.4. Inclusion and Exclusion Criteria

Studies which met the criteria, (1) observational studies including cross-sectional, case-control, and cohort studies, (2) studies that report the prevalence and/or predictors of infant EBF and mixed feeding practices among HIV positive mothers, (3) studies done in Ethiopia, (4) published and unpublished studies at any time, (5) studies that have been written in English language, and 6 studies that reported extractable data to compute the odds ratio of counseling and HIV disclosure status to the spouse, were included in this study. Abstracts, studies without full texts, conference papers, editorials, letters, protocols, program evaluation reports, systematic reviews, trials, and qualitative studies were excluded. Additionally, studies with a high risk of bias/scored less 50% of critical appraisal checklist were excluded.

### 2.5. Outcome Measurement

#### 2.5.1. Exclusive Breastfeeding

The infant receives only breast milk without any other liquids or solids, not even water, except for oral rehydration solution or drops or syrups of vitamins, minerals, or medicines in the first 6 months [39].

#### 2.5.2. Mixed Feeding

It is providing other liquids and/or foods together with breast milk for the infant within six months of age. This could be water, other types of milk, or any type of solid food [39].

### 2.6. Study Screening and Selection

In the beginning, all studies retrieved from the databases were imported to EndNote version 7 citation manager. Next, duplicates were checked and removed. Then, two independent authors (GM and CA) screened the title and the abstracts of retrieved articles. The discrepancies between the authors were solved by discussion and consensus. Consequently, two authors (CA and GM) reviewed the full texts and extracted the data. The first author of the article, year of publication, study area, design, population, region, sample size, extractable data that helps to compute odds ratio of counseling and HIV disclosure status to the spouse, prevalence of EBF, and mixed feeding practices were extracted. Any disagreement between investigators was solved by discussion and repeating of the procedures. Consequently, the data were exported to excel spreadsheets for further analysis.

### 2.7. Quality Assessment

Two independent authors (GM and CA) assessed the quality of the studies using the Joanna Briggs Institute (JBI) quality appraisal checklist [40]. The JBI critical appraisal checklist for analytical cross-sectional studies was employed (Additional file 3). Any disagreement between reviewers was solved by discussion and consensus.

### 2.8. Statistical Analysis

To estimate the national pooled prevalence of EBF and mixed feeding practices among HIV positive mothers, a weighted inverse variance random effects model [41] was used. The publication bias was checked by funnel plot and Egger's regression test. The total percentage of variations between studies due to heterogeneity was assessed by I^2^ statistics [42]. The values of I^2^, 25%, 50%, and 75% represent low, moderate, and high heterogeneity, respectively [42]. The spreadsheet data was exported to Stata version 11 for all statistical analyses. Subgroup analysis by the region was conducted.

### 2.9. Data Synthesis

First, the national pooled prevalence of EBF and mixed feeding among HIV positive mothers were performed separately. Second, the regional pooled prevalence of EBF and mixed feeding practices were done. Third, the pooled OR of counseling and HIV disclosure status to the spouse was performed separately. To compute OR, primarily, number of EBF and mixed feeding practices among counseled and noncounseled mothers were extracted from the cross tabulation of the included studies. In addition, the number of EBF and mixed feeding practices were also extracted from mothers who disclose/not disclose their status to the spouse. Then, the extracted data was exported from excel spreadsheet to Stata version 11 to analyze the pooled OR of counseling and HIV disclosure status to the spouse. Finally, the Preferred Reporting Items of Systematic Review and Meta-Analysis (PRISMA guideline) was used to report the findings of this systematic review and meta-analysis [43].

## 3. Result

### 3.1. Search Results

**Figure 1:**
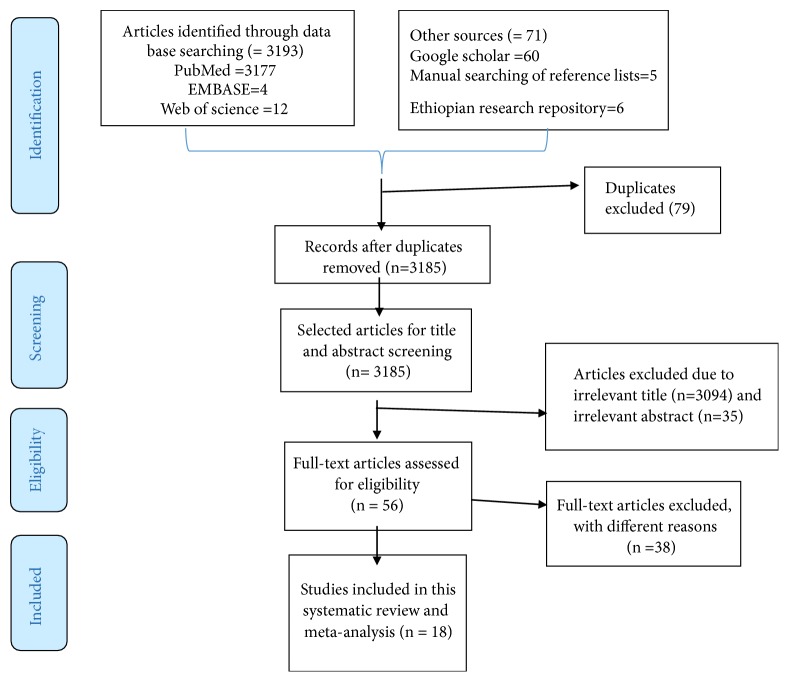
A PRISMA flow diagram of articles screening and process of selection.

A total of 3264 articles were retrieved from different databases of which 3177 were from PubMed, 60 from Google Scholar, 12 from the Web of Science, 4 from EMBASES, 6 from the Ethiopian university research repositories, and 5 from reference lists of the included studies. However, 79 articles were removed due to duplicates, 3129 due to the irrelevant titles and abstracts, 10 due to the study designs, and 20 due to study areas. Finally, 18 articles were included in this systematic review and meta-analysis (Figure 1).

### 3.2. Characteristics of Included Studies

In this study, a total of 18 studies [21–38] with a sample size of 4,844 were included. Of which, five studies [22, 23, 30, 33, 35] were conducted in Amhara region, five [25, 26, 31, 34, 38] in Oromia, four [21, 28, 29, 37] in Addis Abeba, two [24, 32] in Tigray, and two [27, 36] in Southern Nations, Nationalities and Peoples Regional State (SNNPRS). All included studies were conducted with cross-sectional study design. The highest prevalence of exclusive breastfeeding practice among HIV positive mothers was reported in Oromia region 96.6% (95% CI: 93.66,99.54) [26] and the lowest in Addis Abeba 13.4% (95% CI: 8.91, 17.89) [37]. The detailed characteristics of the included studies were described in (Table 1).

### 3.3. Quality of Included Studies

All studies [21–38] were assessed with the JBI quality appraisal checklist for cross-sectional studies [40], and none of them were excluded after assessment (Additional file 4).

### 3.4. Meta-Analysis

**Figure 2:**
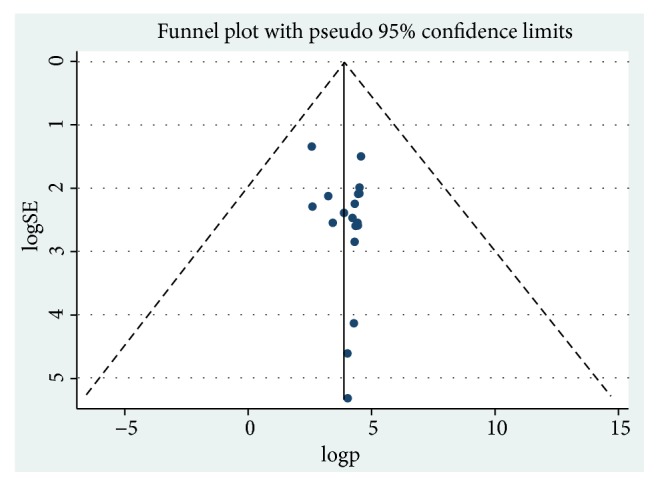
Funnel plot for publication bias, logp, or LNP(log of proportion) represented in the x-axis and standard error of log proportion in the y-axis.

Publication bias was not observed as the funnel plot was symmetrical with visual inspection, and Egger's test value was 0.32 (Figure 2).

### 3.5. Prevalence of Exclusive Breastfeeding Practices

**Figure 3:**
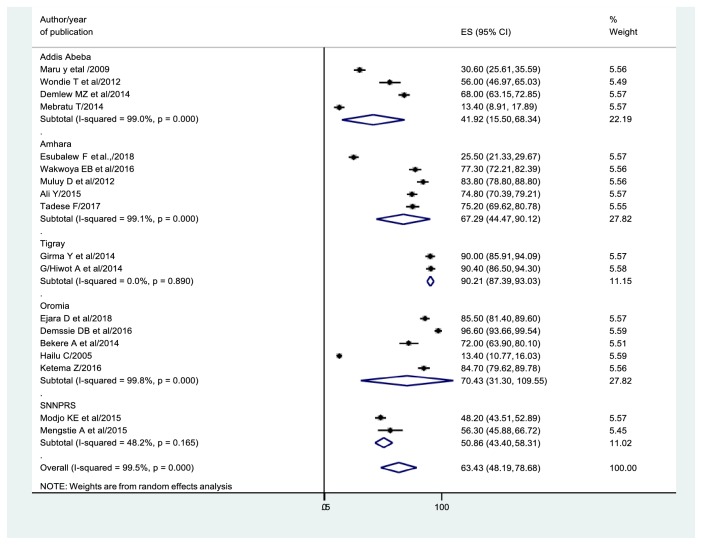
Forest plot of the prevalence EBF with 95% CI. The midpoint and the length of each segment showed the prevalence and 95% CI, respectively. The diamond shape showed the combined prevalence of each region.

On the whole, 18 studies [21–38] were considered to estimate the national prevalence of EBF. Consequently, the overall pooled prevalence of EBF in the current study was 63.43% (95% CI: 48.19, 78.68) (Figure 3).

In the subgroup analysis by the region, the highest prevalence of EBF practices was observed in Tigray 90.12% (95% CI: 87.39, 93.03), and the lowest in Addis Ababa 41.92% (95% CI: 15.50, 68.34). Moreover, the prevalence of EBF practices in other regions includes Oromia (70.43%), Amhara (67.29%), and SNNPRS (50.88%). The detailed descriptions were illustrated in Figure 3.

### 3.6. Characteristics of Studies That Report Mixed Feeding Practices

Out of 18 studies, fifteen [21, 23–27, 30–38] were reporting the prevalence of mixed feeding practices, of which two [21, 37] were conducted in Addis Abeba, four [23, 30, 33, 35] in Amhara region, two [24, 32] in Tigray, five [25, 26, 31, 34, 38] in Oromia, and two [27, 36] in SNNPRS (Table 2).

### 3.7. Prevalence of Mixed Feeding Practices

**Figure 4:**
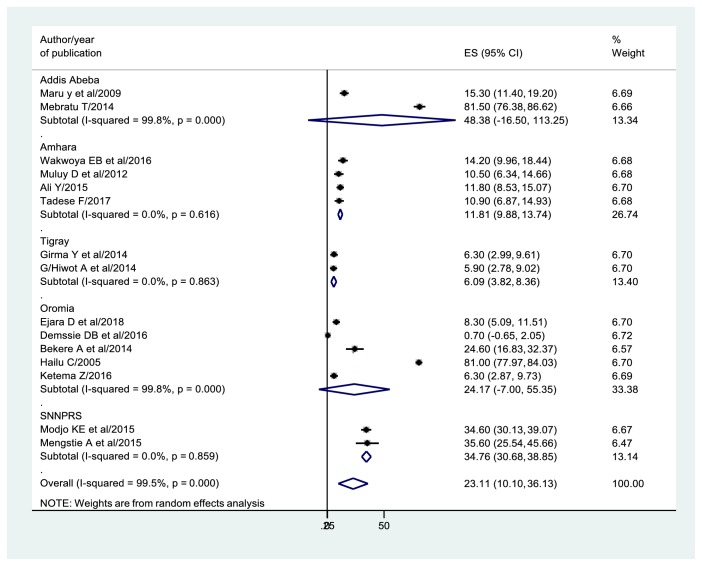
Forest plot of the prevalence of mixed feeding by region with 95% CI. The midpoint and the length of each segment revealed the prevalence and 95% CI of each study whereas the diamond shape showed the combined prevalence.

In this study, the overall pooled prevalence of mixed feeding practices among HIV positive mothers was 23.11% (95% CI: 10.10, 36.13) (Figure 4).

In the subgroup analysis by the region, the highest pooled prevalence of mixed feeding was observed in Addis Ababa (48.38%), followed by SNNPRS (34.76%), Oromia (24.17%), Amhara (11.81%), and Tigray (6.09%) (Figure 4).

### 3.8. The Association between Counseling and Exclusive Breastfeeding Practices

The studies reported different predictors which significantly associated with infant feeding practices of HIV positive mothers of which, counseling on feeding options is the most frequently reported predictors.

**Figure 5:**
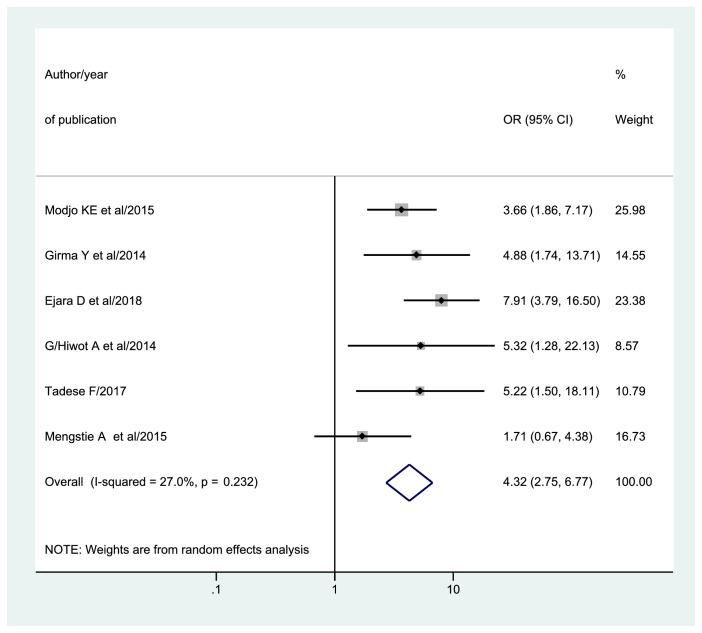
Forest plot of odds ratio of counseling with 95%CI. The midpoint and the length of each segment revealed OR and 95%CI. The diamond shape showed pooled OR.

In this systematic review and meta-analysis, six studies [24, 25, 27, 32, 35, 36] reported data that help to calculate an odds ratio of counseling on feeding options. Consequently, the association between EBF and counseling on feeding options was determined. Therefore, the overall pooled odds ratio of exclusive breastfeeding practices among HIV positive mothers who had been counseled on feeding options was 4.32 (95% CI: 2.75, 6.77) (Figure 5).

### 3.9. The Association between HIV Disclosure to the Spouse and EBF Practices

**Figure 6:**
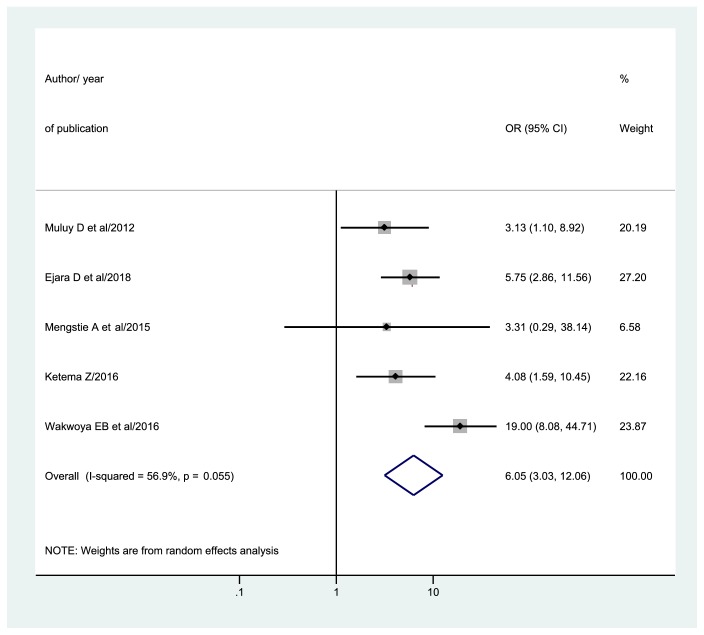
Forest plot of odds ratio of HIV disclosure status to the spouse with 95% CI. The midpoint and the length of each segment showed OR and 95% CI, respectively. The diamond shape revealed pooled OR.

Five studies [23, 25, 30, 36, 38] reported extractable data for determining the association between HIV status disclosure to the spouse and exclusive breastfeeding practices. Following that, the overall pooled odds ratio (OR) of exclusive breastfeeding practices among HIV positive mothers who disclosed their HIV status to the spouse was 6.05 (95% CI: 3.03, 12.06) (Figure 6).

## 4. Discussion

This systematic review and meta-analysis aimed to estimate the national pooled prevalence of exclusive breastfeeding and mixed feeding practices and of its association with counseling and HIV disclosure status to the spouse. The overall pooled prevalence of EBF and mixed feeding practices among HIV positive mothers were 63.43% and 23.11%, respectively. Counseling on feeding options and HIV disclosure status to the spouse were significantly associated with exclusive breast feedings practices.

Regarding the prevalence of EBF practices, the result is in line with the study conducted in Kenya (57.7%) [44], Zambia (40%) [45], Sudan (78%) [46], and Southwestern Nigeria (61%) [47]. On the other hand, the result was higher than two studies conducted in Uganda (24%) [48] and (28%) [49], four studies conducted in South Africa, (35.6%) [50], (30.9%) [50, 51] and (11%) [52], Nigeria (9%) [53], and India (30.6%) [54], but lower than that of the study conducted in Kenya (92%) [55]. The possible reason for the high prevalence of EBF in Ethiopia could be (1) breastfeeding in Ethiopia was considered as a cultural practice and (2) according to EDHS 2016 report, there was high fear of stigma and discrimination in the region except in Addis Ababa [56]. Different studies revealed that high fear of stigma and discrimination was significantly associated with high odds of EBF. For instance, one study conducted in Nigeria revealed a high proportion of mothers practiced exclusive breastfeeding due to fear of stigmatization [57]. (3) The third reason for EBF may be low socioeconomic status in Ethiopia; in this case, the only option of feeding will be breast milk for their infant.

On the other hand, parents from other countries perceived that breast milk alone is not an enough source of nutrition. For instance, in South Africa, there was a belief that breast milk is not enough for their infant [58]. In addition, another study in South Africa revealed that about one-third of the women were introducing other fluids within the first 3 days after birth [59]. Similarly, one study conducted in Uganda revealed that there was a belief that EBF as “not enough” or “even harmful” [60].

Concerning mixed feeding practices, the result was in line with the study done in Nigeria (13%) [47] but lower than the study done in India (43%) [61] and South Africa (61%) [52]. On the other hand, it was higher than studies conducted in southwest Nigeria (4%) [53] and Sudan (4%) [46].

Subgroup analysis revealed that there was a significant variation among regions. In subgroup analysis, the lowest prevalence of EBF practices was observed in Addis Ababa. This variation could be (1) low fear of discrimination in Addis Ababa compared to other regions. According to the 2016 EDHS report, in Addis Ababa there was low fear of discrimination (18%) [56]. This could result in early switching of breastfeeding to formula milk since there will be minimal fear of discrimination when they stop breastfeeding. For instance a study done in Nigeria revealed that a high fear of stigmatization was significantly associated with a high prevalence of exclusive breastfeeding practices [47]. (2) Another possible justification could be the economic status of mothers. As compared to other regions, in Addis Ababa, there is a good living condition in terms of the accessibility of a variety of foods for the infant. One study revealed that regions with lack of funds and poor hygienic conditions were reasons for exclusive breastfeeding [61]. Another study also showed that cultural attitudes, levels of income, and affordability of food were significantly associated with EBF [62].

Concerning predictors, in this systematic review and meta-analysis, HIV positive mothers who have been counseled on feeding options were exclusively breastfeeding their infants nearly four times more likely as compared to none counseled mothers. This finding was in line with the study done in Nigeria [47, 63], Lesotho [64], and Botswana [65].

This was due to the fact that counseled mothers on feeding options would have higher knowledge and awareness on feeding options and PMTCT compared to noncounseled mother [65]. In addition, they would have knowledge of the benefits of EBF and the risks of mixed feedings.

Another factor that was significantly associated with EBF practice was HIV disclosure to the spouse. Even though there are a number of predictors of EBF practices, HIV status disclosure to the spouse is prominent. In this study, we found that the prevalence of exclusive breastfeeding practices among HIV positive mothers who disclose their status to the spouse was nearly six times more likely than mothers who did not disclose their status. This finding was in agreement with the study conducted in Nigeria [63]. The possible reason would be if mothers disclose their HIV status to the spouse, she might get a good family care. For instance, decreased workload, nutritional support, good adherence to ART, and exclusive breastfeeding encouragement were some of the reasons for disclosure [66–68]. Moreover, disclosing HIV status to the spouse prevents mixed feeding [69]. This is due to the fact that HIV positive mothers who disclose their status to the partner will get adequate care, support, and time for breastfeeding [70].

## 5. Conclusion

Most mothers report exclusive breastfeeding, but there are still almost a quarter of mothers who mix feed. Counseling on feeding options and HIV disclosure to the spouse were significantly associated with exclusive breastfeeding practices. Therefore, counseling on feeding options and HIV disclosure status to the spouse should be strengthened.

### 5.1. Limitation of the Study

Even though the this systematic review and meta-analysis aimed to estimate the national prevalence of EBF and mixed feeding practices and its association with counseling and HIV disclosure status, it is not representative for all regions as data was not found in Afar, Somali, Benshangul-Gumuz, Gambella, and Harari. This study also did not determine other factors.

## Figures and Tables

**Table 1:** General characteristics of included studies that reported the prevalence and/predictors of exclusive breast feeding among HIV positive mothers (n=18).

Author/year of publication	Region	Study design	Study area	Populations	Sample size	No. of outcome	Prevalence (%)	Quality
Maru y et al./2009 [21]	Addis Ababa	Cross-sectional	Addis Ababa	Young infant	327	100	30.6	Low risk
Esubalew F et al./2018 [22]	Amhara	Cross-sectional	Gondar Hospital	6-18 months	420	107	25.5	Low risk
Wakwoya EB et al./2016 [23]	Amhara	cross-sectional	Debre Markos Hospital	< 2 yrs.	260	201	77.3	Low risk
Girma Y et al./2014 [24]	Tigray	Cross-sectional	Mekelle Town	< 2 yrs.	207	187	90	Low risk
Ejara D et al./2018 [25]	Oromia	Cross-sectional	Bishoftu towns	<18 months	283	242	85.5	Low risk
Demssie DB et al./2016 [26]	Oromia	Cross-sectional	Shashemene Referral Hospital	<2 yrs.	146	141	96.6	Low risk
Modjo KE et al./2015 [27]	SNNPRS	Cross-sectional	SNNPRS Hospital	<2 yrs.	436	210	48.2	Low risk
Wondie T et al./2012 [28]	Addis Ababa	Cross-sectional	Addis Ababa	<12 months	116	65	56	Low risk
Demlew MZ et al./2014 [29]	Addis Ababa	Cross-sectional	Addis Ababa	<2 yrs.	356	240	68	Low risk
Muluy D et al./2012 [30]	Amhara	Cross-sectional	Gondar health center	<2 yrs.	209	175	83.8	Low risk
Bekere A et al./2014 [31]	Oromia	Cross-sectional	West Oromia	0-6 months	118	85	72	Low risk
G/Hiwot A et al./2014 [32]	Tigray	Cross-sectional	central zone	<2 yrs.	219	198	90.4	Low risk
Ali Y/2015 [33]	Amhara	Cross-sectional	Wollo Zone	2-11 months	373	279	74.8	Low risk
Hailu C/2005 [34]	Oromia	Cross-sectional	Jima town	12 months	643	86	13.4	Low risk
Tadese F/2017 [35]	Amhara	Cross-sectional	Bahir Dar town	12 months	230	173	75.2	Low risk
Mengstie A et al./2015 [36]	SNNPRS	Cross-sectional	SNNPRS hospital	<2 yrs.	87	49	56.3	Low risk
Mebratu T/2014 [37]	Addis Ababa	Cross-sectional	Addis Ababa city	12 months	221	30	13.4	Low risk
Ketema Z/2016 [38]	Oromia	Cross-sectional	Adama health facility	12 months	193	164	84.7	Low risk

*Note*: outcome refers to the number of HIV positive mothers who exclusively breastfeed.

**Table 2:** General characteristics of included studies that reported the prevalence and/predictors of mixed feeding practices among HIV positive mothers (n=15).

Author/year of publication	Study area	Region	Study design	Population	Sample size	Number of outcome	Prevalence (%)	Quality
Maru y et al./2009 [21]	Addis Ababa	Addis Ababa	Cross-sectional	Young infant	327	50	15.30	Low risk
Wakwoya EB et al./2016 [23]	Debre Markos Hospital	Amhara	Cross-sectional	< 2 yrs.	260	37	14.20	Low risk
Girma Y et al./2014 [24]	Mekelle Town	Tigray	Cross-sectional	< 2 yrs.	207	13	6.30	Low risk
Ejara D et al./2018 [25]	Bishoftu town	Oromia	Cross-sectional	<18 months	283	23	8.30	Low risk
Demssie DB et al./2016 [26]	Shashemene Referral Hospital	Oromia	Cross-sectional	<2 yrs.	146	1	0.70	Low risk
Modjo KE et al./2015 [27]	SNNPRS Hospital	SNNPRS	Cross-sectional	<2 yrs.	436	151	34.60	Low risk
Muluy D et al./2012 [30]	Gondar H/c	Amhara	Cross-sectional	<2 yrs.	209	22	10.50	Low risk
Bekere A et al./2014 [31]	West Oromia	Oromia	Cross-sectional	0-6 months	118	29	24.60	Low risk
G/Hiwot A et al./2014 [32]	Central zone	Tigray	Cross-sectional	<2 yrs.	219	13	5.90	Low risk
Ali Y/2015 [33]	Wollo Zone	Amhara	Cross-sectional	2-11 months	373	44	11.80	Low risk
Hailu C/2005 [34]	Jima town	Oromia	Cross-sectional	<12 months	643	521	81.00	Low risk
Tadese F/2017 [35]	Bahir Dar town	Amhara	Cross-sectional	<12 months	230	25	10.90	Low risk
Mengstie A et al./2015 [36]	SNNPRS hospital	SNNPRS	Cross-sectional	<2 yrs.	87	31	35.60	Low risk
Mebratu T/2014 [37]	Addis Ababa city	Addis Ababa	Cross-sectional	12 months	221	180	81.50	Low risk
Ketema Z/2016 [38]	Adama health facility	Oromia	Cross-sectional	12 months	193	12	6.30	Low risk

*Note*: outcome refers to the number of HIV positive mothers who practiced mixed feedings.

## Data Availability

All data generated or analyzed during study are included in this systematic review and meta-analysis.

## Abbreviations

AIDS: Acquired Immunodeficiency Syndrome
ANC: Antenatal Care
AOR: Adjusted Odds Ratio
ART: Antiretroviral Therapy
EBF: Exclusive Breastfeeding
HIV: Human Immune Deficiency Virus
MF: Mixed Feeding
PMTCT: Prevention of Mother-to-Child Transmission
SNNPRS: Southern Nations, Nationalities and Peoples Region State.

## Supplementary Materials

Supplementary 1
* Additional File 1*.* File name*: Additional file 1.* Title*: Preferred Reporting Items for Systematic Review and Meta-Analysis (PRISMA) guideline check list.* Description of data*: the PRISMA guideline contains a 26 checklist items which had been used to report the finding of this study.

Supplementary 2
* Additional File 2*.* File name*: Additional file 2.* Title*: PubMed searching string.* Description of data*: To retrieve articles from the electronic databases, we have used searching terms which are attached as additional file 2.

Supplementary 3
* Additional File 3*.* File name*: Additional file 3.* Title*: JBI critical appraisal checklist for analytical cross-sectional studies.* Description of data*: 8 items of JBI critical appraisal checklist for analytical cross-sectional studies were employed to assess the quality of included study.

Supplementary 4
* Additional File 4*.* File name*: Additional file 4.* Title*: quality assessment result.* Description of data*: critical appraisal result of 18 studies.
