# Supplementary figures and images for: Infant Feeding Practices of HIV Positive Mothers and Its Association with Counseling and HIV Disclosure Status in Ethiopia: A Systematic Review and Meta-Analysis
Source: AIDS Res Treat. 2019 Aug 1;2019:3862098. doi: 10.1155/2019/3862098 (PMC6699255; doi:10.1155/2019/3862098)

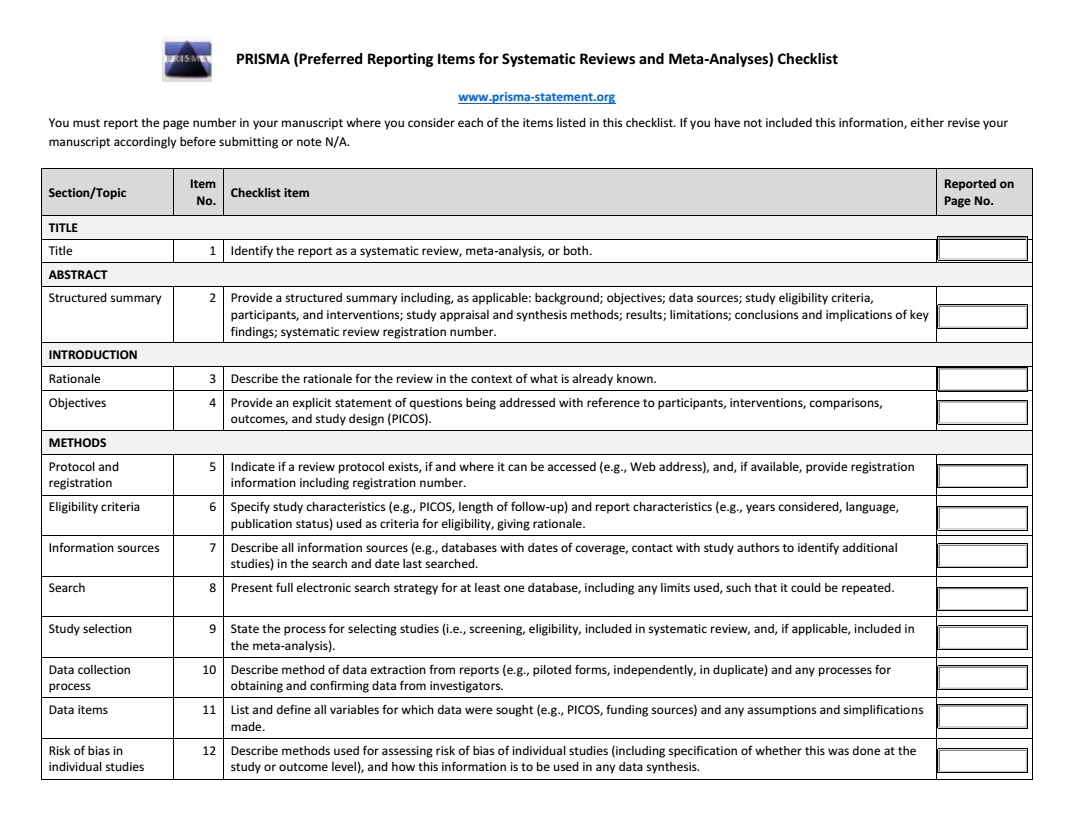


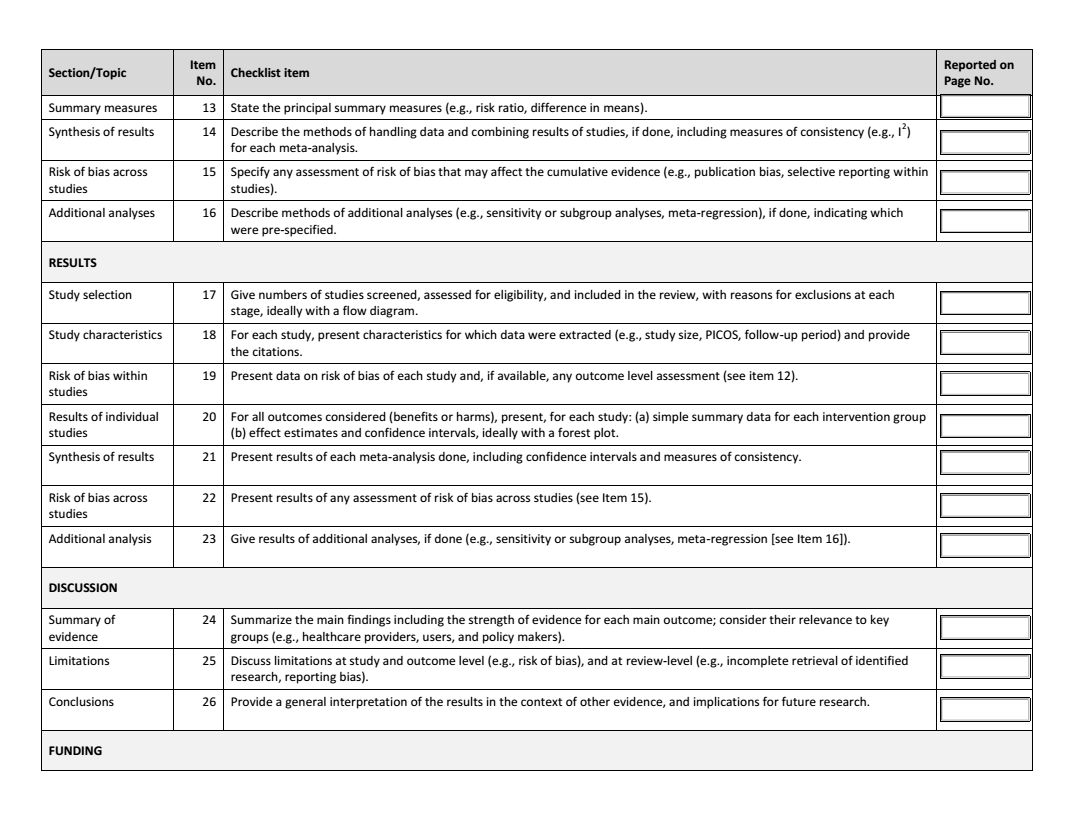

Supplement: Supplementary 1 — Additional File 1. File name: Additional file 1. Title: Preferred Reporting Items for Systematic Review and Meta-Analysis (PRISMA) guideline check list. Description of data: the PRISMA guideline contains a 26 checklist items which had been used to report the finding of this study. [file 3862098.f1.docx]

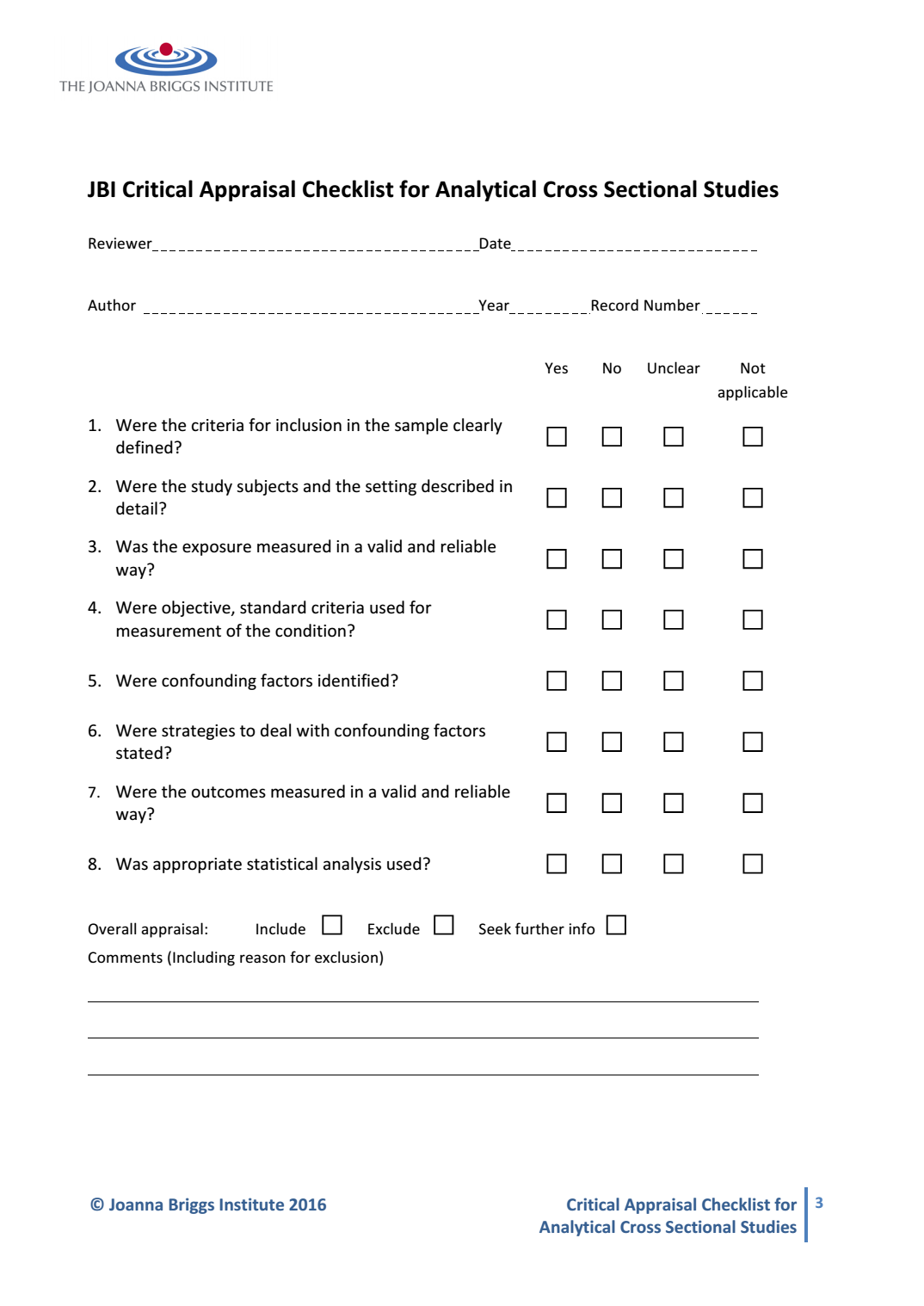

Supplement: Supplementary 3 — Additional File 3. File name: Additional file 3. Title: JBI critical appraisal checklist for analytical cross-sectional studies. Description of data: 8 items of JBI critical appraisal checklist for analytical cross-sectional studies were employed to assess the quality of included study. [file 3862098.f3.docx]
