# Supplementary material for: Infant Feeding Practices of HIV Positive Mothers and Its Association with Counseling and HIV Disclosure Status in Ethiopia: A Systematic Review and Meta-Analysis
Source: AIDS Res Treat. 2019 Aug 1;2019:3862098. doi: 10.1155/2019/3862098 (PMC6699255; doi:10.1155/2019/3862098)
Supplement: Supplementary 2 — Additional File 2. File name: Additional file 2. Title: PubMed searching string. Description of data: To retrieve articles from the electronic databases, we have used searching terms which are attached as additional file 2. [file 3862098.f2.docx]

((infant feeding practices) OR (infant feeding practices[MeSH Terms]) OR (child feeding practices) OR (child feeding practices[MeSH Terms]) OR (young infant feeding practices) OR (young infant feeding practices[MeSH Terms]) OR (exclusive breast feeding practices) OR (exclusive breast feeding practices[MeSH Terms]) OR (exclusive replacement feeding practices) OR (exclusive replacement feeding practices[MeSH Terms]) OR (mixed feeding practices) OR (mixed feeding practices[MeSH Terms]) AND (HIV positive mothers) OR (HIV positive mothers[MeSH Terms]) OR (HIV infected mothers) OR (HIV infected mothers[MeSH Terms]) OR (HIV exposed infants) OR (HIV exposed infants[MeSH Terms]) OR (HIV infected infants) OR (HIV infected infants[MeSH Terms]) AND (predictors) OR (risk factors[MeSH Terms]) OR (associated factors) OR (associated factors[MeSH Terms]) AND (Ethiopia))
