# Supplementary material for: Infant Feeding Practices of HIV Positive Mothers and Its Association with Counseling and HIV Disclosure Status in Ethiopia: A Systematic Review and Meta-Analysis
Source: AIDS Res Treat. 2019 Aug 1;2019:3862098. doi: 10.1155/2019/3862098 (PMC6699255; doi:10.1155/2019/3862098)
Supplement: Supplementary 4 — Additional File 4. File name: Additional file 4. Title: quality assessment result. Description of data: critical appraisal result of 18 studies. [file 3862098.f4.docx]

| Articles | Critical appraisal criteria for cross-sectional studies | | | | | | | | | | | | | | | | | | | | | | | | Total score |
| --- | --- | --- | --- | --- | --- | --- | --- | --- | --- | --- | --- | --- | --- | --- | --- | --- | --- | --- | --- | --- | --- | --- | --- | --- | --- |
|  | Were the criteria for inclusion in the sample clearly defined? | | | Were the study subjects and the setting described in detail? | | | Was the exposure measured in valid and reliable way? | | | Were objective, standard criteria used for measurement of the condition | | | Were confounding factors identified? | | | Were strategies to deal with confounding factors stated? | | | Were the outcomes measured in a valid and reliable way? | | | Was appropriate statistical analysis used? | | |  |
|  | yes | No | NA | Yes | No | NA | Yes | No | NA | Yes | No | NA | Yes | No | NA | Yes | No | NA | Yes | No | NA | Yes | No | NA |  |
| Maru y et al /2009 (36) | √ |  |  | √ |  |  | √ |  |  | √ |  |  |  | × |  |  | × |  | √ |  |  | √ |  |  | 75% |
| Wakwoya EB et al/2016(41) | √ |  |  | √ |  |  |  | × |  | √ |  |  | √ |  |  | √ |  |  | √ |  |  | √ |  |  | 87.5% |
| Girma Y et al/2014(33) | √ |  |  | √ |  |  | √ |  |  | √ |  |  |  | × |  |  | × |  | √ |  |  | √ |  |  | 75% |
| Ejara D et al/2018(31) | √ |  |  | √ |  |  | √ |  |  | √ |  |  |  | × |  | × |  |  | √ |  |  | √ |  |  | 75% |
| Demssie DB et al/2016(30) | √ |  |  | √ |  |  |  | × |  | √ |  |  |  | × |  | × |  |  | √ |  |  | √ |  |  | 62.5% |
| Modjo KE et al/2015(38) | √ |  |  | √ |  |  | √ |  |  | √ |  |  | √ |  |  | × |  |  | √ |  |  | √ |  |  | 87.5% |
| Muluy D et al/2012(39) | √ |  |  | √ |  |  | √ |  |  | √ |  |  |  | × |  | × |  |  | √ |  |  | √ |  |  | 75% |
| Bekere A et al/2014(28) | √ |  |  | √ |  |  |  | × |  | √ |  |  |  | × |  | × |  |  | √ |  |  | √ |  |  | 62.5% |
| G/Hiwot A et al/2014(27) | √ |  |  | √ |  |  | √ |  |  | √ |  |  | √ |  |  | × |  |  | √ |  |  | √ |  |  | 87.5% |
| Ali Y/2015(44) | √ |  |  |  | × |  | √ |  |  | √ |  |  |  | × |  | × |  |  | √ |  |  | √ |  |  | 62.5% |
| Hailu C/2005(34) | √ |  |  | √ |  |  | √ |  |  | √ |  |  | √ |  |  | × |  |  | √ |  |  | √ |  |  | 87.5% |
| Tadese F/2017(40) | √ |  |  | √ |  |  |  | × |  | √ |  |  |  | × |  | × |  |  | √ |  |  | √ |  |  | 62.5% |
| Mengstie A et al/2015(43) | √ |  |  | √ |  |  |  | × |  | √ |  |  |  | × |  | × |  |  | √ |  |  | √ |  |  | 62.5% |
| Mebratu T/2014(37) | √ |  |  | √ |  |  |  | × |  | √ |  |  |  | × |  | × |  |  | √ |  |  | √ |  |  | 62.5% |
| Ketema Z/2016(35) | √ |  |  |  | × |  |  | × |  | √ |  |  | √ |  |  | × |  |  | √ |  |  | √ |  |  | 62.5% |
| Esubalew F et al.,/2018(32) | √ |  |  | √ |  |  | √ |  |  | √ |  |  | √ |  |  | × |  |  | √ |  |  | √ |  |  | 87.5% |
| Wondie T et al/2012(42) | √ |  |  | √ |  |  |  | × |  | √ |  |  | √ |  |  | × |  |  | √ |  |  | √ |  |  | 75% |
| Demlew MZ et al/2014(29) | √ |  |  | √ |  |  |  | × |  | √ |  |  | √ |  |  | × |  |  | √ |  |  | √ |  |  | 75% |
|  |  | | |  | | |  | | |  | | |  | | |  | | |  | | |  | | |  |
